# Editorial: Role of starters on the safety of fermented food products

**DOI:** 10.3389/fmicb.2023.1260403

**Published:** 2023-07-28

**Authors:** Marta Laranjo

**Affiliations:** MED-Mediterranean Institute for Agriculture, Environment and Development, CHANGE-Global Change and Sustainability Institute, Institute for Advanced Studies and Research, Universidade de Évora, Pólo da Mitra, Évora, Portugal

**Keywords:** starters, food safety, microbiome, foodborne pathogens, metagenomics, metabolomics, antimicrobial resistance

Fermented Foods undergo fermentation processes that confer them specific organoleptic properties. Fermentation is a method as old as time to preserve foods, long before the onset of refrigeration or pasteurization, but also a technological procedure to modify raw materials, enhancing the organoleptic properties and transforming them into new foods. Good millennia old examples are bread, wine, and beer. Generally, fermentations may be natural or they may be conducted by microorganisms specifically added for that purpose.

These microorganisms are known as starters or starter cultures, which are individual or mixed microbial cultures used in known concentrations to promote and conduct the fermentation of food products. The use of starters may contribute to enhance organoleptic characteristics, and to the standardization of food products, but also to their safety.

Food safety can be defined as the hygiene procedures undertaken to preserve the quality of food products and prevent their contamination. Food safety deals with the handling, preparation, and storage of food to prevent foodborne diseases.

The aim of this Research Topic was to address the role of starter cultures on the quality and safety of fermented foods.

Starter cultures are mainly used in fermented foods for their ability to promote food safety, mainly due to their antimicrobial activity against spoilage microorganisms and foodborne pathogens, such as *Salmonella* spp., *Listeria monocytogenes*, verocytotoxigenic *Escherichia coli* (VTEC), or *Staphylococcus aureus*, among others. Starters may also play a decisive role on the quality of fermented foods, ensuring product standardization and uniform manufacturing batches. Moreover, they can contribute to the development or enhancement of fermented foods sensory properties.

This Research Topic focused on studies dealing with the role of starters in food safety, covering: the microbiota involved in food fermentations; the selection of technological, protective, and probiotic starter cultures and safety related concerns; metagenomics and metabolomics of fermented foods; the effects of technological procedures in the microbioma of fermented food products; and the traditional and ethnic fermented food products, their microbiota and manufacturing processes.

Of the submitted manuscripts, four have been selected to be part of this Research Topic. Submitting authors come from three different countries, namely Belgium, China, and Spain.

Austrich-Comas et al. evaluated the control of *L. monocytogenes* in chicken dry-fermented sausages with bioprotective starter cultures and high-pressure processing (HPP). Their results evidenced that strategies combining adequate fermentation temperature, and HPP with the use of bioprotective starter cultures may be effective against *L. monocytogenes* contaminated chicken sausages.

Shi et al. explored the technological and safety properties of *Kocuria rhizophila* isolates from traditional ethnic Chinese dry-cured hams. The selected *K. rhizophila* isolates are promising starter candidates for meat fermentation due to their functional and safety properties. Nevertheless, the authors evidenced the need for further study to assess the effect of the selected starters on the sensory qualities of cured meats.

Díaz-Muñoz et al. studied the curing of cocoa beans. The authors used Amplicon Sequence Variant (ASV) analysis, a straightforward microbiological technique, that allowed the reliable and sensitive follow-up of starter microbial strains inoculated in the cocoa pulp-bean mass throughout the cocoa fermentation process. Moreover, starter culture-initiated cocoa fermentation processes presented an enhanced production of desired metabolites, namely volatile organic compounds (VOCs).

Finally, Li et al. investigated the fermentation characteristics of *Lactococcus lactis* subsp. *lactis* isolated from naturally fermented dairy products and screened for potential starter cultures. The authors identified two isolates, which showed a good fermentation capacity and yielded good sensory profiles, making them useful potential starter cultures. Both produced fermented milk with a good malt and nut flavor, mainly due to the presence of 3-methyl butanal and 3-methyl-2-butanone.

We are pleased to introduce this Research Topic, which includes four manuscripts that provide new insights regarding the role of starters on the safety of fermented foods and wish that the readers of *Frontiers in Microbiology* find this topic of relevance and importance to their research.

## Author contributions

ML: Writing–original draft, Writing–review and editing.

